# Indirect Multicultural Experiences: A Multidimensional Journey to Inspire Self-Change

**DOI:** 10.3390/bs15010053

**Published:** 2025-01-08

**Authors:** Jun Zhang, Guanglan Rong, Wenxi Du, Yan Bao

**Affiliations:** 1 College of International Studies, Southwest University, Chongqing 400715, China; jackson@swu.edu.cn (J.Z.); rgl151600@email.swu.cn (G.R.); 2 Westa College, Southwest University, Chongqing 400715, China; 3 Center for Studies of Education and Psychology of Minorities in Southwest China, Southwest University, Chongqing 400715, China; 4 Faculty of Psychology, Southwest University, Chongqing 400715, China

**Keywords:** indirect multicultural experiences, self-change, positive emotion, meaning seeking, subjective and objective social class

## Abstract

Self-change is crucial for driving both individual growth and societal progress. Based on schema theory, this study proposes that indirect multicultural experiences may be an effective pathway to promote self-change, with positive emotions and meaning seeking serving as mediators, and social class as a moderator. To test this hypothesis, an online survey was conducted with 1627 participants aged 18–55 years, using scales to assess indirect multicultural experiences, self-change, positive emotions, meaning seeking, and both subjective and objective social classes. The results indicated that indirect multicultural experiences promote self-change not only by enhancing positive emotions but also by fostering meaning seeking. Additionally, social class moderated the relationship between indirect multicultural experiences and self-change, with subjective social class playing a more pronounced role than objective social class. Individuals with higher subjective social class overall benefited more from indirect multicultural experiences. These findings enhance our understanding of the mechanisms behind self-change and suggest that interventions aiming to foster personal growth and self-improvement can benefit from encouraging diverse cultural exposures, focusing on enhancing individuals’ subjective sense of social class.

## 1. Introduction

Self-change, a key aspect of personal growth, has demonstrably positive effects on individuals and society (Kahn & Isen, 1993). Previous research has explored the consequences of self-change, demonstrating that positive self-change broadens self-image, leading to enhanced self-esteem, coherence, and control (Kahn & Isen, 1993; Stephan & Sedikides, 2024), and can even allow socially excluded people to regain a sense of belonging through self-change and to gain social connections (Richman et al., 2015). The present study will focus on the antecedents of self-change, that is to say, it will examine factors that influence individuals to embark on self-transformation. According to schema theory, encountering new cultures may compel individuals to modify their existing schema, create new ones, and integrate these experiences into their cognitive structures (Shea & Wulf, 2005). This process of multicultural contact, leading to a state of long-term ‘cognitive unfreezing’ (Crisp & Turner, 2011; Sparkman et al., 2016), may foster openness to new self-concepts. In other words, multicultural experiences may serve as catalysts for self-change, prompting individuals to embark on transformative journeys. This study attempts to investigate the mechanisms in this transformation process with the aim of revealing effective strategies for personal growth.

### 1.1. Indirect Multicultural Experiences and Self-Change

Indirect multicultural experiences (IMCE) refer to instances in which individuals are exposed to elements or individuals from foreign cultures without engaging in direct interaction (Aytug et al., 2018; Bao et al., 2024; Maddux et al., 2009). These experiences encompass activities such as watching international films, listening to global music, trying different cuisines, or reading literature from other cultures (Aytug et al., 2018; Bao et al., 2024; Maddux et al., 2009). The rapid advancement of globalization and the internet has significantly increased the accessibility and prevalence of such experiences, enabling individuals to engage with diverse cultural content without traveling abroad (Kahn & Isen, 1993; Lu et al., 2017). Emerging studies suggest that these experiences can significantly affect individuals’ cognitive processes and influence their behaviors (Aytug et al., 2018; Bao et al., 2024; Maddux et al., 2021).

According to schema theory, exposure to new cultures prompts individuals to modify existing schemas, construct new ones, and integrate novel experiences into their cognitive frameworks (Crisp & Turner, 2011). This process not only broadens individuals’ self-concepts but also facilitates changes in their self-schemas. For instance, multicultural experience often triggers a state of “cognitive unfreezing”, wherein individuals reduce their reliance on pre-existing schemas and exhibit greater openness to new information (Maddux et al., 2009; Sparkman et al., 2016). This openness is reflected not only in the acceptance of new ideas and perspectives but also in a willingness to redefine and transform their self-concepts (Maddux et al., 2009).

Empirical evidence provides indirect support for this perspective. For example, Maddux et al. (2009) found that immigrants and international students in cross-cultural contexts are more likely to experience changes in their self-concepts. Moreover, research indicates that individuals with multicultural experiences tend to exhibit higher self-concept clarity (Tadmor et al., 2012a), which, in turn, is associated with a greater willingness to engage in self-change (Butzer & Kuiper, 2006). Consequently, it is reasonable to infer that individuals with more exposure to foreign cultures are more likely to be motivated to undergo self-change. In short, indirect multicultural experiences may promote self-change.

### 1.2. Indirect Multicultural Experiences and Self-Change: The Mediating Role of Positive Emotion

Positive emotion is defined as an individual’s subjective experience of pleasure and joy (Shiota et al., 2021). Research has found that interaction with a new culture promotes changes in individuals’ emotions, thus enabling individuals to be more proactive in adapting to new cultures (Consedine et al., 2014; De Leersnyder et al., 2011). In addition, a multicultural experience can break the traditional way of thinking and enhance the flexibility of individual cognition (DiMaggio, 1997). Individuals with greater cognitive flexibility are more likely to approach problems from diverse perspectives. This cognitive adaptability often involves cognitive reappraisal, a process that focuses on emotion regulation by reframing negative thoughts. That is to say, changes in an individual’s cognition can also regulate their emotion (McRae, 2016) and this process has been found to increase an individual’s experience of positive emotions (Ochsner & Gross, 2008). Therefore, we propose that multicultural experiences may increase positive emotions.

According to the Broaden and Build Theory of Positive Emotion, individuals with more positive emotions have greater motivation for self-change (Slotter & Walsh, 2017). It suggests that positive emotions allow individuals to expand their perspectives and broaden their horizons (Slotter & Walsh, 2017) and this helps people to discover and build significant personal resources (Steger et al., 2008). With these resources, they may seize opportunities and improve themselves in the face of life’s challenges. Empirical studies have shown that individuals who experience more positive emotions are more open to new experiences (Kahn & Isen, 1993), have higher levels of psychological well-being (Fredrickson, 2011), and are more aware of the need for self-improvement (Breines & Chen, 2012). Thus, we hypothesize that positive emotion may promote self-change.

In summary, we propose the hypothesis: indirect multicultural experience may enhance self-change by promoting positive emotion.

### 1.3. Indirect Multicultural Experiences and Self-Change: The Mediating Role of Meaning Seeking

Meaning seeking is defined as the process of discovering and constructing meaning by analyzing and assessing self-value (Steger et al., 2006). Research has shown that meaning seeking is positively correlated with openness to new experiences (Kashdan et al., 2009). For example, it has been confirmed that adults who are receptive to new ideas are more likely to seek and find meaning in their lives (Işik & Uzbe, 2015). Additionally, as previously mentioned, a growing body of research suggests that multicultural experiences, including indirect exposure to different cultures, challenge existing cognitive frameworks and promote a greater willingness to embrace new ideas and worldviews (Endicott et al., 2003; Leung et al., 2008; Maddux et al., 2009; Sparkman et al., 2016). Based on these findings, we propose that indirect multicultural experiences may facilitate meaning seeking.

Meaning in life is one of the most important factors for human beings to withstand adversity in life (Russo-Netzer et al., 2021). When people face adversity, seeking meaning in life helps soothe the soul and motivates people to face difficulties and challenges bravely. Similarly, meaning seeking can stimulate an individual’s inner strength (Park, 2010), which often drives them to carry out practical action (Box et al., 2019). It can also help individuals gain new experiences of meaning (Park, 2010), in other words, in the process of meaning seeking, individuals continue to develop new understandings of themselves and their lives, find new directions in life, and therefore become more satisfied with their lives (Steger & Kashdan, 2007). Along this line, meaning seeking may promote self-change.

In conclusion, we propose the following hypothesis: indirect multicultural experience may enhance self-change by promoting meaning seeking.

### 1.4. Indirect Multicultural Experiences and Self-Change: Subjective and Objective Social Class as Moderator

Social class refers to groups of people’s resources and their perceived rank within the social hierarchy (Kraus et al., 2009). Social class is divided into objective and subjective social class, with the former consisting of indicators such as income level and educational attainment, and the latter defined as a kind of perception of one’s social class (Kraus et al., 2009). Numerous studies have emphasized the important role of social class in facilitating self-change. One study found that people of high social class enjoy richer social resources and will focus on self-development (Lee et al., 2018), which is the main component of self-change. Furthermore, the higher the social class, the easier it is to generate positive emotions such as self-orientated satisfaction, pride, and entertainment (Piff & Moskowitz, 2018). In addition, social class has a strong impact on a person’s sense of self; people in low social classes tend to be less confident in themselves and more likely to be dependent on or focused on others (Hooker & Algoe, 2022), and this may diminish an individual’s focus on self, thus reducing their willingness to change themselves. Based on the above research, we hypothesize that social class may moderate the relationship between indirect multicultural experiences and self-change. Given the unclear direction, we do not make specific predictions and remain open to exploring the moderating effect of social class.

### 1.5. The Current Research

**Figure 1:**
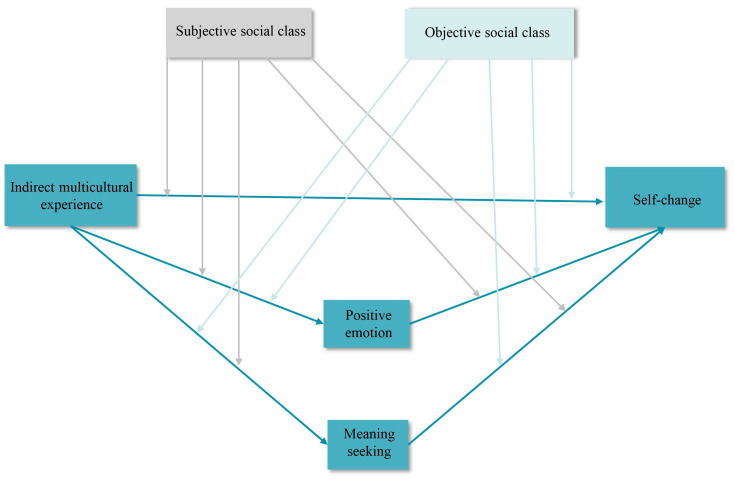
Moderated mediation model of the relationship between indirect multicultural experiences and self-change.

Drawing on schema theory and prior research literature, we propose the moderated mediation model (see Figure 1) to explore the relationship between indirect multicultural experiences and self-change. Specifically, we suppose that the effects of indirect multicultural experiences on self-change are mediated by positive emotions and meaning seeking. And subjective and objective social class may play a moderating role in these relationships. To test these hypotheses, we conducted a questionnaire survey using a large sample from China.

## 2. Methods

### 2.1. Participants

A total of 1645 participants completed the survey online through the Wen Juanxing platform, a Chinese website that specializes in online questionnaires. Eighteen participants were excluded for obvious perfunctory answers (e.g., ‘999 years old’). The final number of valid questionnaires was 1627, of which 1185 (72.8%) were male and 442 (27.2%) were female. The age range was 18–55 years (*M* = 24.32 years, *SD* = 4.47). The study received ethical approval from the local institutional ethics committee.

### 2.2. Measures

#### 2.2.1. Indirect Multicultural Experience

We made minor changes to the multicultural experience item in the Multicultural Experience Assessment scale (Aytug et al., 2018) to measure individual indirect multicultural experiences. Typical items included ‘I often watch various foreign films and videos’ and ‘I often listen to songs in foreign languages’. A total of 9 items were asked on a 7-point Likert scale (from 1 = disagree strongly to 7 = agree strongly). All items were summed and averaged, with higher scores indicating richer indirect multicultural experiences. The Cronbach’s alpha coefficient for the scale in this study was 0.89.

#### 2.2.2. Positive Emotion

The Positive Affect Subscale (Watson et al., 1988) was used to assess the emotional state of individuals. The subscale consists of 10 items, with typical questions such as ‘interested’ and ‘strong’. The participants used a 5-point Likert scale (from 1 = very slightly or not at all to 5 = very much) to indicate the degree to which their current mood matched the topic. All items were summed and averaged, with higher scores indicating a more positive current mood of the individual. The Cronbach’s alpha coefficient for the scale in this study was 0.92.

#### 2.2.3. Meaning Seeking

The meaning-seeking subscale (Steger et al., 2008) was used to measure the extent to which participants were actively seeking meaning or purpose in their lives. The scale consists of 4 items, such as ‘I am searching for a purpose or mission in my life’ and ‘I am searching for meaning in my life’. The items are scored on a 5-point Likert scale (1 = not at all to 5 = very much). The scores for all items were summed to determine the mean, with higher scores indicating greater meaning-seeking tendencies in individuals. The Cronbach’s alpha coefficient for this scale in this study was 0.85.

#### 2.2.4. Self-Change

The Motivation for Self-Change Scale (Lickel et al., 2014) was used to assess the extent to which the participants were willing to change themselves. A total of 4 items were included, such as ‘I felt the urge to be a better person’ and ‘I felt the need to change myself after the event’. The scale was scored on a 5-point Likert scale (from 1 = disagree to 5 = agree). The scores for all items were summed for the mean, with higher scores indicating a stronger willingness of the individual to change themselves. The Cronbach’s alpha coefficient for this scale in this study was 0.82.

#### 2.2.5. Subjective Social Class

The MacArthur Subjective Social Status Scale (Adler & Ostrove, 1999) was used to assess participants’ perceptions of their social class. Participants were shown an image of a ladder consisting of 10 rungs symbolizing different income, educational, and occupational statuses in China. The top rung represented those whose living situation was the most privileged, while the bottom rung represented those whose living situation was the worst. Participants indicated their position on the ladder by choosing a corresponding number; the higher the number, the higher they considered their social class.

#### 2.2.6. Objective Social Class

The objective social class of the subjects was represented by their level of education, which was measured by the question ‘Regarding your level of education, please select the appropriate option’. There were six options: 1 = ‘graduate student (Master’s or PhD)’, 2 = ‘undergraduate’, 3 = ‘college’, 4 = ‘high school or junior college’, 5 = ‘junior high school’, and 6 = ‘primary schools or below’. We reverse scored this, with higher values indicating higher levels of education, i.e., higher objective social class.

#### 2.2.7. Control Variable

Because this study explored the relationship between indirect multicultural experiences and self-change, we asked the participants to report their overseas duration (how long they had been abroad: 1 = never been abroad, 2 = half a month, 3 = one month, 4 = two months, 5 = half a year, 6 = one year, and 7 = two years or more) to exclude confounding effects from direct multicultural experiences.

### 2.3. Analysis and Results

#### 2.3.1. Common Method Biases Test

To avoid the effects of common method bias, we strictly controlled the response mode, response statements, and anonymity in answering the questionnaires during the administration process. After the questionnaires were collected, exploratory factor analysis (Zhou, 2004) was conducted on all the items using Harman’s one-way test, and the results showed that there were five factors with eigenvalues greater than 1, and the explanatory rate of the first common factor was 32.04%, which was less than the critical value of 40%. Therefore, there was no significant common method bias in this study.

#### 2.3.2. Descriptive Statistics and Correlations

The descriptive statistics and correlation results for each variable are presented in Table 1. The results indicated that indirect multicultural experiences were significantly positively correlated with positive emotions, meaning seeking, and self-change, which set the stage for subsequent analyses. Participants’ gender, age, and overseas duration were correlated with some of the core variables in this study and were therefore used as control variables.

#### 2.3.3. Indirect Multicultural Experience Positively Predicts Self-Change

Stepwise regression analysis was conducted with self-change as the dependent variable. The results showed (Table 2 for details) that indirect multicultural experience significantly and positively predicted self-change (*β* = 0.18, *t* = 7.39, *p* < 0.001). In the second step, after adding the control variables (gender, age, and length of time abroad), the indirect multicultural experience still significantly and positively predicted self-change (*β* = 0.29, *t* = 10.92, *p* < 0.001).

#### 2.3.4. Parallel Mediating Effects of Positive Emotions and Meaning Seeking

**Figure 2:**
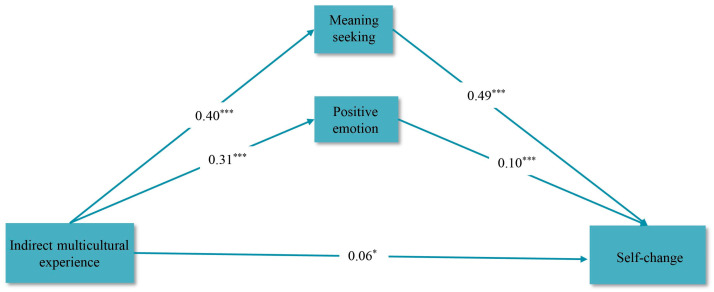
Parallel intermediation model diagram (standardized coefficients). Note: * *p* < 0.05, *** *p* < 0.001 (two tails).

Model 4 of the SPSS 26.0 macro PROCESS v3.5 (Hayes, 2018) was used to test the mediating role of positive emotions and meaning seeking between indirect multicultural experiences and self-change. The results are shown in Table 3. Step 1 indicated that indirect multicultural experiences significantly positively predicted positive emotions (*β* = 0.31, *t* = 12.26, 95% CI = [0.26, 0.36], *p* < 0.001). Step 2 showed that indirect multicultural experiences positively predicted meaning seeking (*β* = 0.40, *t* = 15.6, 95% CI = [0.35, 0.45], *p* < 0.001). Step 3 showed that indirect multicultural experiences significantly positively predicted self-change (*β* = 0.06, *t* = 2.45, 95% CI = [0.01, 0.11], *p* = 0.01). Moreover, positive emotions and meaning seeking also significantly positively predicted self-change (*β* = 0.10, *t* = 4.01, 95% CI = [0.05, 0.15], *p* < 0.001; *β* = 0.49, *t* = 19.95, 95% CI = [0.44, 0.54], *p* < 0.001). This suggested that positive emotions and meaning seeking simultaneously play a partial mediating role in the relationship between indirect multicultural experiences and self-change (Figure 2).

#### 2.3.5. Moderating Effects of Subjective and Objective Social Status

**Figure 3:**
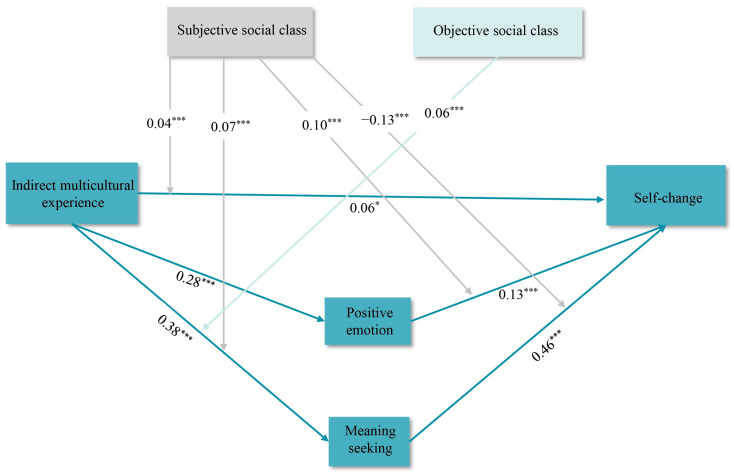
Moderated mediation model (standardized coefficients). Note: * *p* < 0.05, *** *p* < 0.001 (two tails).

The moderating roles of subjective and objective social class in the parallel mediation model were tested using Model 76 of the SPSS 26.0 macro program PROCESS v3.5, and the results are presented in Table 4. The interaction term between indirect multicultural experience and subjective social class positively predicted meaning seeking (*β* = 0.07, *t* = 3.35, 95% CI = [0.03, 0.10], *p* = 0.001) and self-change (*β* = 0.04, *t* = 2.29, 95% CI = [0.01, 0.08], *p* = 0.02). The interaction term between indirect multicultural experience and objective social class significantly and positively predicted positive emotions (*β* = 0.06, *t* = 2.79, 95% CI = [0.02, 0.10], *p* = 0.01). The interaction term between positive emotions and subjective social class significantly positively predicted self-change (*β* = 0.10, *t* = 4.47, 95% CI = [0.05, 0.14], *p* < 0.001), while the interaction term between meaning seeking and subjective social class significantly and negatively predicted self-change (*β* = −0.13, *t* = −5.35, 95% CI = [−0.18, −0.08], *p* < 0.001). This finding suggested that subjective social class moderated the four pathways of “indirect multicultural experience—meaning seeking”, “indirect multicultural experience—elf-change”, “meaning seeking—self-change”, and “positive emotions—self-change” and that objective social class moderated the pathway of “indirect multicultural experience—positive emotions” (see Figure 3).

**Figure 4:**
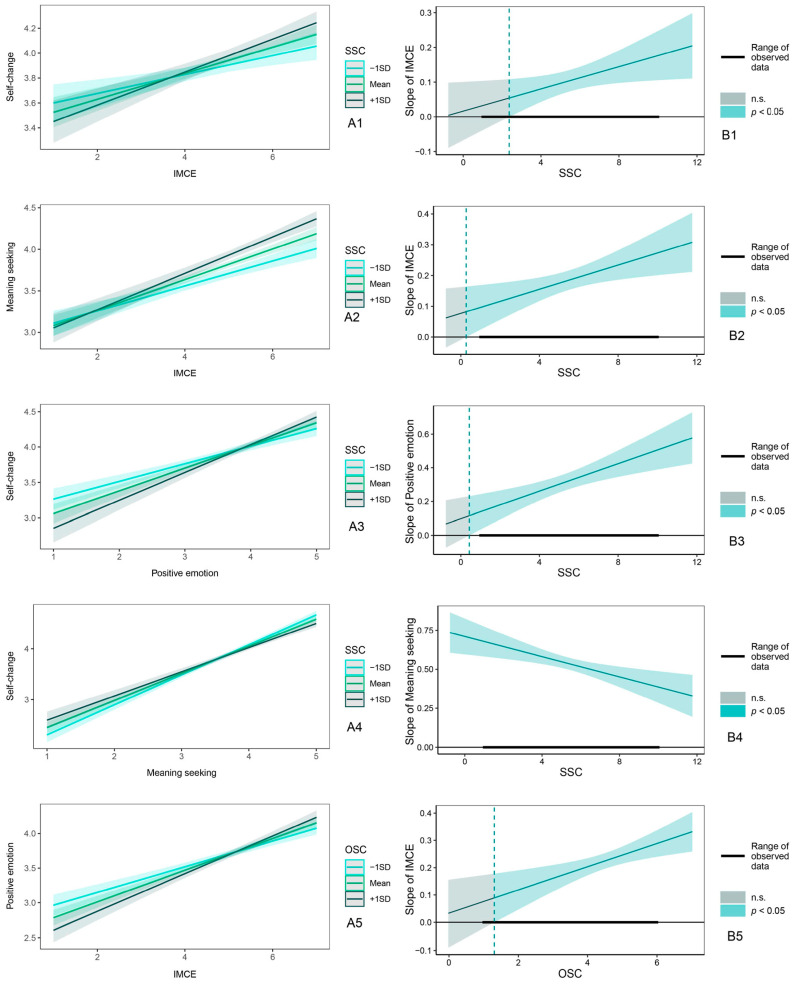
Simple slope plots of moderating effects. Panel (**A**) (point-estimation method): (**A1**) SSC moderates “IMCE—Self-change”; (**A2**) SSC moderates “IMCE—Meaning seeking”; (**A3**) SSC moderates “Positive emotion—Self-change”; (**A4**) SSC moderates “Meaning seeking—Self-change”; (**A5**) OSC moderates “IMCE—Positive emotion”. Panel (**B**) (J-N method): (**B1**) SSC moderates “IMCE—Self-change”; (**B2**) SSC moderates “IMCE—Meaning seeking”; (**B3**) SSC moderates “Positive emotion—Self-change”; (**B4**) SSC moderates “Meaning seeking—Self-change”; (**B5**) OSC moderates “IMCE—Positive emotion”.

To further explore the moderating effect, we conducted five simple slope test analyses using the pick-a-point approach. The results are shown in Figure 4. When individuals’ subjective social class was one standard deviation above the mean (+1 SD), indirect multicultural experiences significantly positively predicted self-change and meaning seeking and had a strong promoting effect on both; when individuals’ subjective social class was at the mean, indirect multicultural experiences significantly predicted self-change and meaning seeking, but the effect was weaker; and when individuals’ subjective social class was one standard deviation below the mean (−1 SD), indirect multicultural experiences still significantly predicted self-change and meaning seeking, but the effect was the weakest (Figure 4(A1,A2)). Similarly, when individuals’ subjective social class was one standard deviation above the mean (+1 SD), positive emotions had a stronger effect on self-change. The facilitating effect of positive emotions on self-change diminished as subjective class declined (Figure 4(A3)). However, as subjective social class increased, the facilitating effect of meaning seeking on self-change diminished (i.e., for higher classes, the role of meaning seeking in promoting self-change was weaker than that of the lower classes; Figure 4(A4)). In addition, indirect multicultural experiences had the strongest effect on positive emotions when individuals’ objective social class was one standard deviation above the mean (+1 SD); when the objective class was at the mean, indirect multicultural experiences had a weaker effect on positive emotions; and when the objective class was one standard deviation below the mean (−1 SD), indirect multicultural experiences were still a significant predictor of positive emotions, but at this point, the effect was the weakest (Figure 4(A5)). In summary, the effects of subjective social class were stronger and more widespread. The objective class, on the other hand, was relatively weak.

In addition, based on the simple slope results of the pick-a-point approach above, we found that the facilitation effects all seemed to increase with increasing class. We therefore conducted additional analyses to explore the regions with significant moderating effects using the Johnson–Neyman (J–N) method (Figure 4). It was found that when subjective social class was higher than 2.36, the confidence interval of the simple slope line excluded 0, and this slope line increased along the X-axis, indicating that subjective social class positively moderated the relationship between indirect multicultural experiences and self-change, i.e., the positive effect of IMCE on self-change became stronger as the subjective class increased; in other words, the IMCE no longer promoted self-change when the subjective class was lower than 2.36 (Figure 4(B1)). When subjective social class is greater than 0.27, the confidence interval of the simple slope line excludes 0, and the slope line increases along the X-axis, suggesting that subjective class positively moderates the relationship between IMCE and meaning seeking; in other words, indirect multicultural experiences will no longer promote meaning seeking when subjective class is lower than 0.27 (Figure 4(B2)). When subjective social class is greater than 0.43, the 95% CI of the simple slope does not contain 0, and the promotion of self-change by positive emotions becomes stronger as subjective class increases, again illustrating the positive moderating effect of subjective social class on the relationship between positive emotions and self-change (Figure 4(B3)). Figure 4(B4) shows that the confidence intervals of the simple slope lines exclude 0, and the slope lines are always above and decreasing along the X-axis, indicating that subjective social class negatively moderates the relationship between meaning seeking and self-change. In addition, when the objective social class is greater than 1.31, the 95% CI of the simple slope line excludes 0, and the slope line increases along the X-axis, suggesting that the objective class positively moderates the relationship between indirect multicultural experiences and positive emotions (Figure 4(B5)); in other words, when the objective class is lower than 1.31, indirect multicultural experiences will no longer promote positive emotions.

## 3. Discussion

Through a large-sample survey, this study constructed and validated a moderated mediation model of “from indirect multicultural experiences to self-change”. The results indicate that indirect multicultural experiences are significantly positively correlated with self-change, and this relationship is mediated by positive emotions and meaning-seeking motivations. Specifically, richer indirect multicultural experiences bring stronger positive emotional experiences and heightened motivations for meaning seeking, resulting in greater self-change. Furthermore, the study also reveals the moderating effects of both objective and subjective social class on the above mediation path. Notably, compared to objective social class, subjective social class exerts a more pronounced moderating influence on self-change. In other words, subjective social class more readily influences self-change. These findings provide a novel perspective for deepening the understanding of the relationship between multicultural experiences and self-change.

### 3.1. The Positive Association Between Indirect Multicultural Experiences and Self-Change

The results of this study demonstrate a positive correlation between indirect multicultural experiences and self-change. This finding is consistent with previous research, which has indicated that a multicultural environment serves as a pivotal factor in promoting individual self-development (Dimesinova & Moldakhmetov, 2012). Specifically, multicultural experiences not only enhance individuals’ insight and stimulate creativity (Leung et al., 2008) but also improve cognitive flexibility (Tadmor et al., 2012a), thereby fostering the development of an open-minded and growth-oriented mindset (Aytug et al., 2018). Individuals who frequently engage with multicultural elements tend to think from multiple perspectives, engage in continuous self-reflection, and pursue higher levels of self-actualization (Fowers & Davidov, 2006). In line with symbolic interactionism theory, these findings can be further understood. This theory posits that individuals construct meaning and self-concept through interactions with symbols in their social environment (Ukasoanya, 2014). In the context of globalization, individuals often encounter diverse cultural elements—such as foreign songs, books, and movies—through international products. This exposure goes beyond passive information reception; it involves actively engaging with cultural symbols, which helps individuals form new cognitive frameworks. These frameworks, in turn, influence and reshape their existing self-concept (Johnson & Johnson, 2009; John-Steiner & Mahn, 1996), potentially leading to self-change (Ro et al., 2013).

### 3.2. The Mediating Role of Positive Emotions

Consistent with our hypothesis, the current study found that indirect multicultural experiences promote self-change by enhancing positive emotions. This finding is supported by previous research, such as Consedine et al. (2014), which pointed out the emergence of emotional adaptation during repeated exposure to new cultures, particularly in multicultural environments where individuals’ emotions may exhibit heightened sensitivity and adaptability. This heightened emotional adaptability is often closely linked to well-being (Berry & Hou, 2016), potentially helping individuals experience more positive emotions. Positive emotions, exemplified by happiness, are widely regarded as the driving force behind sustained individual behavior (Carver & Scheier, 1990) and a critical factor in enhancing one’s action efficacy (Fredrickson, 1998). They serve as vital tools for individuals to achieve psychological growth (Fredrickson, 2011). Research has shown that individuals with abundant positive emotions exhibit greater resilience and adaptability when confronted with challenging or risky situations (Russell, 2003). Those who are highly satisfied with their life tend to actively engage in current life experiences, a process that not only deepens their understanding of the world but also fosters the integration of these experiences with their newly constructed worldview (Youngstrom & Green, 2003), potentially leading to the expansion of self-boundaries in a subtle yet profound manner. Consequently, the positive emotions fostered by indirect multicultural experiences may not only stimulate individuals’ action potential but also provide an internal impetus for their self-development.

### 3.3. The Mediating Role of Meaning Seeking

The current study also found that indirect multicultural experiences promote self-change by fostering meaning seeking. The pursuit of meaning in life is an eternal theme in human life’s journey (Steger et al., 2006), constituting an integral part of human motivational structures (Doğan et al., 2012). Core traits of this motivation, such as openness to ideas and intellectual curiosity (Steger et al., 2006), collectively propel individuals to continually explore and comprehend the deeper meanings of life. Tests on the multicultural personality of international students have revealed significant openness in their multicultural worldview (Yakunina et al., 2012). This not only signifies an acceptance of foreign cultures but also embodies a desire to explore the unknown. Exposure to cultures from different countries or ethnic cities often brings about unprecedented novel experiences (Alhazmi & Kaufmann, 2022), aiding individuals in acquiring new knowledge and understanding. Therefore, individuals with rich multicultural experiences are more likely to adopt a positive and open mindset, deeply seeking meaning in life amidst these novel experiences. As a positive indicator of individual mental health (Steger et al., 2008), meaning seeking is positively correlated with intrinsic motivation and concentration (Steger et al., 2006, 2008). In other words, individuals who actively seek meaning in life demonstrate stronger motivation and sustained focus in their self-endeavors. Additionally, research has shown that meaning seeking prompts individuals to deeply reflect on and question their existing values and life perspectives (Steger et al., 2008). This process may inspire individuals to continuously explore new perspectives and opportunities, and courageously break through their limitations, thereby embarking on a new chapter of personal growth and transformation.

### 3.4. Moderating Effect of Social Class

Within the overall model of multicultural experiences leading to self-change, the moderating effect of social class, particularly subjective social class, is prominent. Subjective social class positively moderates the relationships between “multicultural experiences—elf-change” and “multicultural experiences—meaning seeking”: The higher the subjective social class, the stronger the facilitative effect of multicultural experiences on self-change and meaning seeking. This finding aligns with previous research results (Kraus & Park, 2014; Quon & McGrath, 2014) in that subjective social class fosters individuals’ willingness to engage in self-transformation under the influence of foreign cultural elements. The underlying reason may lie in the high level of openness exhibited by individuals from higher social classes (Lin et al., 2021), who tend to focus on their unique traits, goals, and inner feelings (Boucher, 2021) and believe in their ability to leverage existing resources and conditions for self-improvement (Belmi et al., 2020). In contrast, individuals from lower subjective social classes are particularly sensitive to external environments (Kraus et al., 2009) and often suffer from low self-esteem (Shea & Wulf, 2005) and learned helplessness (Odabasi, 2013), believing that even effort is unlikely to alter their current circumstances, leading to a conservative attitude towards self-change.

Furthermore, subjective social class positively moderates the relationship between “positive emotions and self-change”: The higher the subjective social class, the stronger the facilitative effect of positive emotions on self-change. Individuals with high subjective social class perceive themselves as having control over their lives, which tends to foster high self-esteem (Moradi & Hasan, 2004) and generate positive emotions and life satisfaction (Chen et al., 2016; Orth et al., 2012; Tian et al., 2013). This suggests that individuals are in a good state and perceive opportunities for further pursuit in their environment (Russell, 2003), motivating them to continuously engage in self-improvement to chase new experiences. In contrast, individuals with low subjective social class report lower levels of happiness and life satisfaction (Botha et al., 2018), and anxiety inhibits their pursuit of an ideal life (Serin et al., 2010). Even when experiencing positive emotions, they tend to maintain the status quo rather than actively seeking change for a better life (Gross, 2015). In the relationship between “meaning seeking and self-change”, subjective social class exhibits a negative moderating effect. For individuals from lower social classes, the motivation for meaning seeking significantly enhances their willingness to engage in self-change, while it has no apparent impact on those from higher social classes. This may be attributed to the fact that individuals with high subjective social class possess more resources, enabling them to creatively navigate social events (Kraus et al., 2012), which to some extent diminishes the role of meaning-seeking motivation in triggering self-change. Notably, within the overall model, objective social class plays a weaker role compared to subjective social class, positively moderating only the relationship between ‘indirect multicultural experiences and positive emotions’. This finding aligns with previous research, indicating that subjective social class better captures individuals’ self-perceptions of social status and exerts a stronger predictive power over their motivations and behaviors (Quon & McGrath, 2014).

### 3.5. Implications

This study contributes both theoretically and practically to the field of personal growth by delving into the intricate associations among indirect multicultural experiences, positive emotions, meaning seeking, and self-change. Self-change is a pivotal factor driving individual development (Kahn & Isen, 1993). To our knowledge, this research is the first to reveal indirect multicultural experiences as a potential antecedent of self-change and to identify the mediating roles of positive emotions and meaning seeking in this relationship, thereby enhancing our understanding of the internal mechanisms underpinning the process of self-change. Furthermore, the current study also highlights the significant role of subjective social class in moderating the relationship between indirect multicultural experiences and self-change, with individuals of higher subjective social class benefiting more from these experiences. These findings underscore the importance of fostering a strong sense of subjective social class and integrating this awareness into interventions aimed at promoting personal growth. Lastly, numerous prior studies have shown that multicultural experiences can foster creativity (Hundschell et al., 2022) and reduce group biases (Tadmor et al., 2012b). The current findings, which reveal a significant correlation between multicultural experiences and a stronger willingness to engage in self-change, further broaden the spectrum of positive psychological effects attributed to multicultural experiences.

### 3.6. Future Direction

Future research could be further expanded and refined in several key areas. First, studies could explore how openness to experience influences the relationship between indirect multicultural experiences and self-change. Openness may encourage individuals to seek out multicultural experiences (Matz, 2021) and also facilitate self-change (McCrae, 1996). Future studies could control for openness to assess the robustness of this relationship, or explore its potential role as a moderator or mediator. Second, the correlational design used in the current study does not establish causality. Future research could manipulate indirect multicultural experiences to test for causal effects. Additionally, given that self-change may both result from and drive indirect multicultural experiences, future studies could employ longitudinal designs or bidirectional path models to explore the reciprocal relationship between them. Finally, future research could further distinguish between types of indirect multicultural experiences, particularly in terms of their breadth and depth (Lu et al., 2017; Zhao et al., 2024). We suggest breadth (e.g., exposure to a variety of foreign cultures) may have a more significant impact on self-change than depth (e.g., the duration of exposure to foreign cultures). Additionally, it is important to differentiate between positive and negative indirect multicultural experiences (Carver & Scheier, 1990). Positive experiences may promote self-change, while negative experiences (e.g., involuntary exposure to foreign cultures with distressing content) could lead to resistance or even negative outcomes.

## 4. Conclusions

This study suggests that indirect multicultural experiences may facilitate self-change by enhancing positive emotions and promoting meaning seeking. Moreover, social class, particularly subjective social class, plays a significant moderating role in this process, with individuals of higher subjective social class overall benefiting more from indirect multicultural experiences. These findings provide valuable insights into the psychological mechanisms underlying self-change and emphasize the importance of incorporating multicultural exposure in interventions aimed at fostering personal growth. Future research could further explore these dynamics and investigate how best to apply these findings to enhance individual development.

## Figures and Tables

**Table 1:** Means, standard deviations, and correlation coefficients of variables.

	*M*	*SD*	1	2	3	4	5	6
1. IMCE	4.80	1.19						
2. Positive emotion	3.65	0.74	0.36 ***					
3. Meaning seeking	3.79	0.79	0.31 ***	0.51 ***				
4. Self-change	3.93	0.74	0.18 ***	0.31 ***	0.57 ***			
5. Gender	0.27	0.44	−0.06 *	−0.17 ***	0.06 *	0.20 ***		
6. Age	24.32	4.47	0.07 **	0.10 ***	0.07 **	−0.02	−0.07 **	
7. Overseas duration	3.12	2.22	0.44 ***	0.25 ***	−0.03	−0.13 ***	−0.22 ***	0.08 **

Note: * *p* < 0.05, ** *p* < 0.01, *** *p* < 0.001 (two tails). Gender was dummy-coded: female = 1, male = 0.

**Table 2:** Regression analysis with self-change as dependent variable.

	Step 1				Step 2			
	*B*	*SE*	*β*	*t*	*B*	*SE*	*β*	*t*
IMCE	0.11	0.02	0.18	7.39 ***	0.18	0.02	0.29	10.92 ***
Gender					0.28	0.04	0.17	7.02 ***
Age					0.00	0.00	−0.01	−0.54
Overseas duration					−0.07	0.01	−0.21	−7.99 ***
*R* ^2^	0.03				0.11			
Δ*R*^2^	0.03				0.08			
*F*	54.62 ***				48.80 ***			

Note: *** *p* < 0.001 (two tails).

**Table 3:** Parallel mediation test (standardized coefficients).

	Step 1: Positive Emotion			Step 2: Meaning Seeking			Step 3: Self-Change		
	*SE*	*β*	*t*	*SE*	*β*	*t*	*SE*	*β*	*t*
IMCE	0.03	0.31	12.26 ***	0.03	0.40	15.6 ***	0.02	0.06	2.45 *
Positive emotion							0.03	0.10	4.01 ***
Meaning seeking							0.02	0.49	19.95 ***
Overseas duration	0.03	0.08	3.19 **	0.03	−0.20	−7.66 ***	0.02	−0.12	−5.27 ***
Gender	0.05	−0.13	−5.4 ***	0.05	0.04	1.82	0.05	0.16	7.75 ***
Age	0.00	0.06	2.66 **	0.00	0.06	2.68 **	0.00	−0.05	−2.45 *
*R* ^2^	0.16			0.14			0.37		
*F*	77.51 ***			65.49 ***			156 ***		

Note: * *p* < 0.05, ** *p* < 0.01, *** *p* < 0.001 (two tails).

**Table 4:** Parallel mediation test with moderation (standardized coefficients).

	Step 1: Positive Emotion			Step 2: Meaning Seeking			Step 3: Self-Change		
	*SE*	*β*	*t*	*SE*	*β*	*t*	*SE*	*β*	*t*
IMCE	0.03	0.28	11.15 ***	0.03	0.38	14.69 ***	0.02	0.06	2.64 **
SSC	0.02	0.24	10.23 ***	0.02	0.18	7.38 ***	0.02	0.01	0.33
IMCE × SSC	0.02	0.01	0.71	0.02	0.07	3.35 **	0.02	0.04	2.29 *
OSC	0.02	−0.02	−0.85	0.02	−0.02	−1.02	0.02	0.06	2.92 **
IMCE × OSC	0.02	0.06	2.79 **	0.02	0.01	0.49	0.02	0.00	−0.22
PE							0.03	0.13	4.99 ***
MS							0.02	0.46	18.44 ***
PE × SSC							0.02	0.10	4.47 ***
MS × SSC							0.02	−0.13	−5.35 ***
PE × OSC							0.03	−0.04	−1.58
MS × OSC							0.03	−0.04	−1.24
Overseas duration	0.03	0.04	1.38	0.03	−0.25	−9.28 ***	0.02	−0.13	−5.56 ***
Gender	0.02	−0.11	−5.01 ***	0.02	0.05	2.28 *	0.02	0.16	7.58 ***
Age	0.02	0.03	1.45	0.02	0.04	1.51	0.02	−0.04	−2.12 *
*R* ^2^	0.22			0.17			0.39		
*F*	55.95 ***			42.47 ***			72.84 ***		

Note: * *p* < 0.05, ** *p* < 0.01, *** *p* < 0.001 (two tails). Abbreviations: IMCE = indirect multicultural experiences; SSC = subjective social class; OSC = objective social class; PE = positive emotion; MS = meaning seeking.

## Data Availability

The data presented in this study are openly available in OSF at https://osf.io/5qf8e/ (accessed on 26 October 2024).
